# Marriage of an Epileptic—Murder Committed by Him on the Same Day

**Published:** 1846-02

**Authors:** 


*Marriage of an Epileptic—Murder committed by him on the same day.*—Francis Seveil, aged 20, a shoemaker, had for a number of years been subject to attacks of epilepsy. They commenced from a fall on the ice. The paroxysms, which at first were attended with only slight aberrations of reason, gradually became more serious, and were accompanied with furious mania.

He had served in the 5th light troops from 1838 to 1841, and when off duty, pursued his trade. When attacked during this period, he would seize his hammer, knife, or any instrument at hand, and brandish it in a threatening manner, thus subjecting himself to the jests of his comrades.

When discharged, he returned and determined to marry. The ceremony with his affianced was fixed for the 26th of October, 1841. On the 24th, severe pains of the head came on, and which seemed to him an indication of an approaching attack. He called on the physician, who had secretly treated him for the complaint, and asked that he might be bled—an operation from which he had always derived relief. The physician declined, on the ground that this remedy should not be too frequently employed. On the 26th, a few hours before the marriage, he was bled by another physician, but without a dimunition of the pain.

During the civil, as well as the religious ceremonies of the nuptials, Seveil was sedate and taciturn. He said nothing beyond the simple yes. On leaving the church, he was seized with most excruciating pain in the head, and this was so overpowering, that at the house of his father-in-law he was oblignd to go to bed. The room in which he lay was adjoining that in which the nuptial dinner was spread. Here he was seized with a fit of furious epilepsy, and while the persons with him had run out to obtain ropes to bind him, he rushed naked into the dining room, with a shovel that he snatched up, pursued a female, who fled from him, and knocked her down with a blow on the head. His father-in-law interposed, but he in turn with others, were chased. He then sat down on the ground before the door, grinding the pebbles with his teeth, and finally standing up with a shoemaker's knife in his hand, he burst open the door, exclaiming that he must kill them all. The first person that he met was his father-in-law, and whom he killed on the instant.

This attack continued for three days, so that they had to confine him in a sack. On the 29th, reason returned, but he could only remember the marriage—nothing subsequent—and supposed that he had slept since that time. He was soon transferred to the Asylum at Clement, where he still remains.

Under these circumstances, the guardian of Seveil applied to the Court, for a declaration of the nullity of the marriage, on the ground that the epileptic was not at the time in his sane mind, and could not therefore give a proper consent.

The counsel in favor of this, urged strongly the idea, that attacks of epilepsy are always preceded by gloom and taciturnity, and that the headache was a further proof that the mind was already in a diseased state.

The presumption at last was in favor of mental alienation at the moment of the ceremony.

The court decided for the nullity of the marriage.—*Gazette Des Tribunaux, Jan. 7th,* 1845.—*American Journal of Insanity.*

